# Cochrane plain language summaries are highly heterogeneous with low adherence to the standards

**DOI:** 10.1186/s12874-016-0162-y

**Published:** 2016-05-23

**Authors:** Antonia Jelicic Kadic, Mahir Fidahic, Milan Vujcic, Frano Saric, Ivana Propadalo, Ivana Marelja, Svjetlana Dosenovic, Livia Puljak

**Affiliations:** Cochrane Croatia, University of Split School of Medicine, Soltanska 2, 21000 Split, Croatia; Faculty of Medicine, University of Tuzla, Univerzitetska 1, 75000 Tuzla, Bosnia and Herzegovina

**Keywords:** Cochrane systematic reviews, Plain language summary, Dissemination, Simple language, Adherence, Quality standards

## Abstract

**Background:**

The aim of this study was to analyze whether Cochrane plain language summaries (PLSs) adhere to the Standards for the reporting of Plain Language Summaries in new Cochrane Intervention Reviews (PLEACS).

**Methods:**

A systematic analysis of adherence to the measurable PLEACS items was performed for Cochrane PLSs published from March 2013 to the end of January 2015. Duplicate independent data extraction was performed. An adherence score was calculated for each PLS and for the Cochrane Review Groups (CRGs) that published them.

**Results:**

Of the 1738 analyzed PLSs, not a single one adhered fully to the measured PLEACS items. The highest adherence was found for absence of details of the search strategy (99 % adherence), and the lowest adherence for an item mandating to address quality according to the GRADE system (0.7 % adherence). Overall adherence percentage of PLSs reporting reviews with included studies was 57 %. Different CRGs had a wide range of adherence scores.

**Conclusions:**

Cochrane plain language summaries are highly heterogeneous with a low adherence to the PLEACS standards. Therefore, there is much room for improving the content and consistency of the PLS. A standardization of PLSs is necessary to ensure delivery of proper and consistent information for consumers.

**Electronic supplementary material:**

The online version of this article (doi:10.1186/s12874-016-0162-y) contains supplementary material, which is available to authorized users.

## Background

Systematic reviews are considered the highest level of evidence because they include systematic analysis of literature of a particular research question. As such, systematic reviews make evidence accessible and usable by a busy clinician, and particularly for experts engaged in making clinical guidelines in different fields of medicine. However, systematic reviews may be long and difficult to understand and therefore many readers will access only abstracts to get the key message [1]. In addition to conventional scientific abstracts, Cochrane systematic reviews have a plain language summary (PLS), which is aimed towards general public. The Cochrane PLSs are supposed to be clear, understandable and accessible, especially for the lay persons in particular field of medicine. It would be desirable to write PLSs in a standard format, so the Cochrane has published Standards for the reporting of Plain Language Summaries in new Cochrane Intervention Reviews (PLEACS) [2].

Since all Cochrane reviews are prepared and published in English, the Cochrane has recognized the need to promote evidence-informed health care by publishing its high-quality content in languages other than English. At the moment, there are 13 Cochrane translation teams around the world, managing translations into Chinese (simplified and traditional), Croatian, French, German, Japanese, Korean, Malay, Polish, Portuguese, Russian, Spanish and Tamil [3]. Most of them translate only PLSs. Therefore, a PLS is now an important knowledge translation tool, not only in English, but in numerous languages worldwide [4]. Therefore, it is now more important than ever to ensure high quality and homogeneity of the PLSs, and their adherence to standards.

These standards complement the *Methodological Expectations of Cochrane Intervention Reviews* (MECIR) project, which aims to standardize the production of Cochrane systematic reviews [5]. However, while reading a Cochrane PLS one often finds technical and scientific jargon that may be very difficult for consumers to understand. Additionally, it is unclear what proportion of the Cochrane PLSs is adhering to the PLEACS standards. Therefore, the aim of this study was to analyze heterogeneity of the PLSs and their adherence to the PLEACS guidance for writing PLSs. We hypothesized that the Cochrane PLSs are very heterogeneous considering their structure, length and information they contain, and that majority of them are not written entirely according to the PLEACS.

## Methods

A systematic analysis of the PLSs’ adherence to the measurable items of PLEACS was performed using *a priori* defined research protocol. Cochrane PLSs published from March 2013 to the end of January 2015 were searched. Withdrawn Cochrane reviews were excluded from the study. Six authors extracted data (MV, FS, AJK, MF, IP, IM). Duplicate independent data extraction was performed for each PLS. Independent data extractions were checked for consistency and discrepancies were resolved by another author (SD).

For analysis, we used the latest version of PLEACS, which is currently publicly available; the version 3.0 dated February 28, 2013. The PLEACS contains 14 items, of which 12 are marked as ‘mandatory’ and two as ‘highly desirable’ (PLS9 and PLS11). From those mandatory items, we identified measurable units eligible for independent assessment by reviewers and therefore the collected data do not necessarily correspond to the complete instructions provided in the PLEACS. Since the goal of the study was to assess the PLSs’ heterogeneity, additionally we also analyzed word count of PLSs. Even though the PLEACS say: “It is highly desirable for the PLS to be 400 words and it should be no more than 700 words.“,  this sentence is located within the PLS1, which is marked as mandatory.

Certain elements that are indicated as ‘mandatory’ in the PLEACS were not analyzed, such as PLS4, related to ‘Consistency’, because the way it is described indicates that the key messages of the PLS need to be consistent with the text of review and the Summary of Findings table in the review, and therefore we deemed that this item is not measurable solely by analyzing the PLS.

Apart from the date of publication (month, year) and the Cochrane review group, the following 14 data items were collected from the PLS: 1) Is the title of the systematic review and the title of the PLS the same? (*the title should be restated in plain language*), 2) Number of words (*recommended: 400 words; not more than 700 words*), 3) Did they avoid technical terms and jargon – words that are specifically recommended to be avoided *((outcome, literature, case series, efficacy and effect size) as well as terms that may have slightly different meanings in medicine than in common usage (e.g. local, blinding, control, practice))*?*,* 4) Is PLS narrative or structured (*headings are mandatory*)?, 5) If structured, are the subtitles as recommended (*Review Question, Background, Study Characteristics, Key Results, Quality of Evidence*)?, 6) If structured, how many recommended headings are missing?, 7) Is the search date indicated?, 8) Did they include details of the search strategy (*i.e. databases, search terms should not be included*)?, 9) Are there population details?, 10) Is there information about the number of studies included in the systematic review?, 11) Is there information about the number of participants in included studies?, 12) Are there any complex statistical data used and not explained?, 13) Is quality of the included studies addressed?, and 14) Is quality addressed according to the GRADE system?

For empty systematic reviews data for items 9–14 were not collected. A summary adherence score was calculated for each PLS based on the 14-item scoring system indicated in the Additional file 1, where points were assigned for having mandated elements. Four items (1, 3, 8 and 12) were scored in reverse because for these, certain elements should not be present.

Based on this adherence scoring, each PLS with included studies could have a minimum of 0 points to a maximum of 19 points for adherence to the measured PLEACS items, while empty reviews could have 0-13 points. Overall adherence to the measured PLEACS items of PLSs and Cochrane Review Groups (CRGs) that published them was expressed as a percentage.

Since the latest version of PLEACS, which was used in this study, was published in February 28, 2013, we decided to analyze PLSs published from March 2013 up to the day of the start of this study so that we can analyze whether the consistency of PLSs improves over time.

Descriptive statistics (frequencies and percentages) for each domain were used to gain insight into the proportion of PLSs written according to the measured PLEACS items using Microsoft Excel (Microsoft Corp., Redmond, WA, USA). Correlation between time of publication and adherence score was calculated.  Linear regression analysis was conducted to explore association between time of publication and adherence score using GraphPad Prism (GraphPad Software, La Jolla, CA, USA).

## Results

We found 1799 PLSs published between March 2013 and January 2015, 61 were withdrawn, leaving 1738 PLS for analysis. Of those, there were 176 empty reviews with no studies included. None of the included PLSs was updated subsequently during the analyzed period.

Table 1 indicates how many of the PLS adhered to different PLEACS items that were scored in this study. The most successful adherence was to the item regarding the absence of details of the search strategy (99 % adherence) and absence of unexplained complex statistical data (98 % adherence), while the lowest adherence was observed related to addressing quality according to the GRADE system (0.7 % adherence).

**Table 1:** Number of Cochrane plain language summaries adhering to the PLEACS standards

	PLEACS items	N (%) of PLS adhering to the PLEACS item^a^
1	Title of systematic review restated in plain language	1105 (64)
2	Number of PLSs within recommended word count (400-700)	404 (23)
3	Technical terms and jargon absent	748 (43)
4	PLS structured	626 (36)
5	If structured, subtitles as recommended	217 (35)
6	If not structured as recommended, how many of the 5 recommended subtitles are missing?	1 missing: 141 (34)
		2 missing: 90 (22)
		3 missing: 47 (10)
		4 missing: 40 (10)
		5 missing: 94 (23)
7	Search date indicated	Month and year: 992 (57)
		Year only: 20 (11)
8	Details of search strategy absent	1718 (99)
9	Population details provided	236 (15)
10	Number of studies indicated	1429 (91)
11	Number of participants indicated	1201 (77)
12	Complex statistical data absent	1527 (98)
13	Quality of the studies addressed	875 (56)
14	GRADE system mentioned	11 (0.7)

^a^Items 1–4 and 7–8 calculated for all included PLSs (*N* = 1738), items 9–14 for those that were not empty (*N* = 1562). Item five was calculated only for structured PLSs (*N* = 626) and item six for PLSs not structured as recommended (*N* = 409)

Mean number of words in the PLS was 319 (median 304, range 46-1125). Few PLSs (*N* = 8, 0.5 %) had a higher number of words than the recommended range between 400–700 words. However, a significant portion of the PLS had a lower number of words than the minimum mandated by the PLEACS (*N* = 1326, 76 %).

Quality scoring indicated that overall average quality score of 1562 reviews with included studies was 11 (range: 4–18 points) of maximum 19 points, indicating average adherence of 57 % with the analyzed recommendations. There were 10 (0.6 %) PLSs with adherence of 0–25 %, 307 (20 %) with adherence of 26–50 %, 1051 (67 %) with adherence of 51–75 % and 194 (12.4 %) with adherence of 75–100 %.

Among 176 empty reviews, average quality score for their PLSs was 7.6 (range 3–12) of maximum 13 points, indicating average adherence of empty review PLSs of 59 %. There were 2 (1.1 %) PLSs with adherence of 0–25 %, 32 (18 %) between 26–50 %, 122 (69 %) between 51–75 % and 20 (11 %) with adherence above 76 %.

Not a single PLS completely adhered to the standards.

Analysis of PLSs published by different CRGs showed that PLSs were published by 53 review groups in this period. Their output was very heterogeneous, ranging from publishing 3 to 159 Cochrane reviews during the analyzed 23 months. The range of total adherence percentage for different CRGs was from 43 % (Cochrane Incontinence Group, published 8 PLSs in the analyzed period) to 81 % (Cochrane Oral Health Group, 16 PLSs). A CRG devoted to consumers and communication (Cochrane Consumers and Communications group) had adherence to the PLEACS of 61 % (17 PLSs). We noticed that certain Cochrane review groups consistently use their own format of preparing the PLS, which is different from PLEACS items.

The value of Pearson correlation coefficient between time of publication and adherence score was 0.235, indicating a weak positive association. A linear regression analysis was used to test increase in adherence over time. The slope showed significant difference from zero (p < 0.001) suggesting increase in adherence over time.

## Discussion

This study showed that Cochrane plain language summaries are highly heterogeneous, in terms of length and adherence to recommended standard for their writing. Cochrane is a global non-profit organization, dedicated to producing and promoting credible and accessible health information. The Cochrane thus represents an international gold standard for high quality, trusted information [6]. However, Cochrane systematic reviews are produced by numerous Cochrane review groups, which do not necessarily use the recommended editorial processes. It has already been demonstrated that methods of Cochrane reviews may vary, depending on the Cochrane review group which produced the review [7].

Cochrane plain language summaries were developed as a simple format of presenting key information from Cochrane systematic reviews to patients [8]. The PLS is considered a main building block for dissemination of the review to the end-users of health information. Therefore, it is commendable that there are efforts to develop a standardized language for describing statistical results, based on the effect size and quality of supporting evidence. Producing standardized high-quality PLS may enable more effective dissemination [9]. There are also different ideas on how to improve format of the PLS, based on the information that consumers might want to read [8, 10].

The latest version of the PLEACS standards for preparing the PLS was published on February 28, 2013. Even though there was another version of the PLEACS available before that, in this analysis we included only PLSs that were published after the latest version of the PLEACS. Our analysis indicates that the Cochrane PLSs are widely heterogeneous in terms of length and various items of the PLEACS standards. Standard scientific abstracts of Cochrane reviews all follow the same style. Having the same style and format for the PLSs may help consumers to find quickly information they are looking for in specific parts of a summary. Additionally, it is worth emphasizing that the majority of PLSs were shorter than recommended. Some of them had as little as 46 words – this PLS consists of three short sentences [11]. Such brevity may not necessarily allow proper explanation about what was done in a review and what the results mean to lay audience.

It is important to emphasize that in this manuscript we did not analyze the PLEACS themselves. It is possible that these standards for writing the plain language summaries are not ideal themselves and not evidence-based, but this is another topic. However, if standards exist in an organization, they should be followed uniformly.

## Conclusion

Among 1738 analyzed PLSs, not a single one adhered fully to the measured PLEACS items. Therefore, there is much room for improving the content and consistency of the PLS. To improve consistency of information presented in the Cochrane PLSs and to enhance dissemination of evidence among consumers, Cochrane review groups should closely follow standards for writing simple summaries of Cochrane systematic reviews.

## Additional file

Additional file 1: Summary quality scoring for each Cochrane plain language summary (PLS) based on their analyzed characteristics. (DOCX 14 kb)

## Abbreviations

CRG: cochrane review group
PLEACS: standards for the reporting of plain language summaries in new Cochrane intervention reviews
PLS: plain language summary
