# Supplementary material for: Cochrane plain language summaries are highly heterogeneous with low adherence to the standards
Source: BMC Med Res Methodol. 2016 May 23;16:61. doi: 10.1186/s12874-016-0162-y (PMC4877986; doi:10.1186/s12874-016-0162-y)
Supplement: Additional file 1: — Summary quality scoring for each Cochrane plain language summary (PLS) based on their analyzed characteristics. (DOCX 14 kb) [file 12874_2016_162_MOESM1_ESM.docx]

**Additional file 1**

Summary quality scoring for each Cochrane plain language summary (PLS) based on their analyzed characteristics

| **PLEACS item** | | **Scoring points** |
| --- | --- | --- |
| 1 | Is the title of systematic review and title of the PLS the same? *(reverse scoring)* | Yes – 0 point  No – 1 points |
| 2 | Number of words within recommended range (400-700) | Yes – 1 point  No – 0 points |
| 3 | Technical terms and jargon? *(reverse scoring)* | Yes – 0 points  No – 1 point |
| 4 | Is PLS structured? | Yes – 1 point  No – 0 points |
| 5 | If structured, are subtitles as recommended? | Yes – 1 point  No – 0 points |
| 6 | How many recommended subtitles are missing? | 5 – 0 points  4 – 1 point  3 – 2 points  2 – 3 points  1 – 4 points  0 – 5 points |
| 7 | Search date indicated | Yes – 2 points  Year – 1 point  No – 0 points |
| 8 | Details of search strategy included *(reverse scoring)*? | Yes – 0 point  No – 1 points |
| 9 | Population details? | Yes – 1 point  No – 0 points |
| 10 | Number of studies | Yes – 1 point  No – 0 points |
| 11 | Number of participants | Yes – 1 point  No – 0 points |
| 12 | Complex statistical data present *(reverse scoring)*? | Yes – 0 points  No – 1 point |
| 13 | Quality of the studies addressed | Yes – 1 point  No – 0 points |
| 14 | GRADE system mentioned | Yes – 1 point  No – 0 points |
|  | Total maximum score for reviews with studies included (items 1-14) | 19 |
|  | Total maximum score for empty reviews (items 1-8) | 13 |

Acronyms: GRADE = Grading of Recommendations Assessment, Development and Evaluation, PLEACS = Standards for the reporting of Plain Language Summaries in new Cochrane Intervention Reviews, PLS = plain language summary
